# Expression of Hyaluronan Synthases (*HAS1–3*) and Hyaluronidases (*HYAL1–2*) in Serous Ovarian Carcinomas: Inverse Correlation between *HYAL1 *and Hyaluronan Content

**DOI:** 10.1186/1471-2407-9-143

**Published:** 2009-05-12

**Authors:** Timo K Nykopp, Kirsi Rilla, Reijo Sironen, Markku I Tammi, Raija H Tammi, Kirsi Hämäläinen, Anna-Mari Heikkinen, Marja Komulainen, Veli-Matti Kosma, Maarit Anttila

**Affiliations:** 1 Institute of Clinical Medicine, Pathology and Forensic Medicine, University of Kuopio and Kuopio University Hospital, Kuopio, Finland; 2 Institute of Biomedicine, Department of Anatomy, University of Kuopio, Kuopio, Finland; 3 Department of Obstetrics and Gynecology, University of Kuopio and Kuopio University Hospital, Kuopio, Finland

## Abstract

**Background:**

Hyaluronan, a tumor promoting extracellular matrix polysaccharide, is elevated in malignant epithelial ovarian tumors, and associates with an unfavorable prognosis. To explore possible contributors to the accumulation of hyaluronan, we examined the expression of hyaluronan synthases (*HAS1*, *HAS2 *and *HAS3*) and hyaluronidases (*HYAL1 *and *HYAL2*), correlated with hyaluronidase enzyme activity hyaluronan content and HAS1–3 immunoreactivity.

**Methods:**

Normal ovaries (n = 5) and 34 serous epithelial ovarian tumors, divided into 4 groups: malignant grades 1+2 (n = 10); malignant grade 3 (n = 10); borderline (n = 4) and benign epithelial tumors (n = 10), were analyzed for mRNA by real-time RT-PCR and compared to hyaluronidase activity, hyaluronan staining, and HAS1–3 immunoreactivity in tissue sections of the same specimens.

**Results:**

The levels of *HAS2 *and *HAS3 *mRNA (*HAS1 *was low or absent), were not consistently increased in the carcinomas, and were not significantly correlated with HAS protein or hyaluronan accumulation in individual samples. Instead, the median of *HYAL1 *mRNA level was 69% lower in grade 3 serous ovarian cancers compared to normal ovaries (P = 0.01). The expression of *HYAL1*, but not *HYAL2*, significantly correlated with the enzymatic activity of tissue hyaluronidases (r = 0.5; P = 0.006). An inverse correlation was noted between *HYAL1 *mRNA and the intensity of hyaluronan staining of the corresponding tissue sections (r = -0.4; P = 0.025).

**Conclusion:**

The results indicate that in serous epithelial ovarian malignancies *HAS *expression is not consistently elevated but *HYAL1 *expression is significantly reduced and correlates with the accumulation of hyaluronan. (233 words)

## Background

Because early-stage ovarian cancer produces nonspecific symptoms, the majority of patients continue to present with advanced disease, with limited chances of complete surgical removal of the malignant tissue. The disease often spreads by implantation through the mesothelial surfaces covering the abdominal cavity. One of the suggested contributors for this spreading is the CD44 receptor on cell surface and its ligand, the extracellular matrix polysaccharide hyaluronan [1,2]. We have found a 49-fold increase in the median concentration of hyaluronan in grade 3 ovarian carcinomas, and an 89-fold increase in the corresponding metastases compared to normal ovary. [3] This is consistent with the fact that hyaluronan is an independent, unfavorable prognostic factor in epithelial ovarian cancer [1], and suggests that hyaluronan is involved in the progression of this, and other malignancies [4]. Blocking the accumulation of hyaluronan might offer a new way of fighting against the disease, and defining the causes of the accumulation should provide means for this.

Hyaluronan can be produced in mammals by three hyaluronan synthase isoenzymes: HAS1, HAS2 and HAS3 [5]. HAS mRNA levels often correspond to the rate of hyaluronan synthesis, and are known to influence the content of hyaluronan in transplanted tumors [6]. Therefore, upregulation of HAS expression is a likely contributor to the hyaluronan accumulation in tissues, and promotes tumor growth [7] and metastasis in experimental animals, in particular when coexpressed with hyaluronidase [8].

The tissue content of hyaluronan is balanced by catabolism, which is a more complex process [9]. Hyaluronan in the extracellular matrix can be partially fragmented by hyaluronidase activity or oxygen free radicals, and diffuse away through lymph. Alternatively, hyaluronan can be taken up by adjacent cells and be subject to lysosomal degradation in the tissue of origin [10]. Therefore, the formation of oxygen free radicals, access to lymph, local uptake by cells, and hyaluronidases may each have a contribution to the rate of hyaluronan catabolism. There are 6 hyaluronidases in the human genome, two of them (*HYAL1 *and *HYAL2*) ubiquitous and characterized at protein level [9,11]. The major transcript of *HYAL3 *is enzymatically inactive and appears to have only a supportive role in *HYAL 1 *expression [12]. *HYAL 3 *knockout mice did not display any evidence of hyaluronan accumulation [13]. Very little is known about HYAL4, but its expression is limited, and it might be a chondroitinase rather than hyaluronidase [11,14]. The expression of *SPAM1*-encoded PH20 hyaluronidase is almost exclusively detected in testis and sperm, and shows activity in higher pH. We have shown previously that ovarian tissues show no hyaluronidase activity at pH 7 [3].

In an invasive bladder cancer cell line, blocking of *HYAL1 *expression decreases tumor growth, inhibits tumor infiltration and decreases microvessel density [15]. Increased hyaluronidase expression has also been reported in colon [9] and prostate cancer [16], as well as in breast tumor metastases [17]. In contrast, experimental overexpression of *HYAL1 *in a rat colon carcinoma cell line inhibits tumor growth and generates necrotic tumors [6]. Recent findings have suggested that depending on its concentration, *HYAL1 *can function as a tumor promoter or as a suppressor [16].

We started to determine the mechanism of hyaluronan accumulation in ovarian cancer by analyzing the expression profiles of hyaluronan synthases and hyaluronidases in a clinically defined set of tumors, and found that a significantly decreased *HYAL1 *expression correlates with lower hyaluronidase activity and elevated hyaluronan content of the tumors, while *HAS *expression was not as consistently associated to the accumulation of hyaluronan.

## Methods

### Patients

39 ovarian tissue specimens from 39 patients were divided into 5 groups: normal ovaries (n = 5), serous cystadenomas (n = 10), serous borderline tumors (n = 4), low grade (grades 1+2) (n = 10) and high grade (grade 3) (n = 10) serous epithelial cystadenocarcinomas. The borderline and malignant ovarian tumors were staged according to FIGO (Table 1). The ethical committee of the Kuopio University Hospital has approved the study protocol and patients signed the informed consent.

**Table 1:** Clinicopathological data of the epithelial ovarian tumor samples*

	Tumor type (*n)*		
	
Variable	Malignant	Borderline	Benign
Age at diagnosis^†^	57 (43–83)	66 (20–82)	62 (16–72)
			
Histological type			
Serous	20	4	10
Histological grade			
1	3^‡^		
2	7^‡^		
3	10		
FIGO stage			
I		2	
II	1		
III	13	2	
IV	6		

*In addition to the diseased tissues shown in the table, five samples of normal ovarian tissue were analysed

^† ^medians (range)

^‡ ^In this study grade I and grade II tumors were combined into one subgroup

### Histology

Histological typing and grading was done according to the WHO classification [18] 14. Grade 1 and 2 cancers were combined into one subgroup.

### Tissue samples

Tissue specimens collected in operation room were evaluated and tumor sections were prepared by pathologist. All the samples were collected and handled identically. Aliquots of the tissues were 1) placed in RNAlater^® ^(Ambion, Austin, TX) for mRNA analyses; 2) fixed in 10% buffered formalin and embedded in paraffin; and 3) homogenized in 1 mM sodium EDTA containing 1 mM benzamidine-HCl, 1 mM saccharic acid- 1,4-lactone, 1 mM β-mercaptoethanol, 1 mM iodoacetate, and 0.5% Triton X-100, clarified by centrifugation at 4°C (1,000 × g for 15 min and 10,000 × g for 30 min) and the extracts stored at -70°C until assayed.

### RNA Extraction and cDNA Preparation

The samples were frozen by liquid nitrogen and pulverized under pressure using a stainless steel cylinder and a piston. Total RNA was isolated using Trizol^® ^Reagent (Invitrogen) according to manufacturer's protocol, quantified spectrophotometrically and its integrity confirmed by agarose electrophoresis, based on the appearance of the 18 S and 28 S RNA bands. First strand cDNA was synthesized from 2.5 μg of total RNA using High-Capacity cDNA Archive kit (Applied Biosystems, Foster City, CA) according to manufacturer's protocol in a final volume of 50 μl.

### Quantitative real-time RT-PCR

The PCR primers and fluorogenic probes for all target genes (*HYAL1*, *HYAL2*, *HAS1–3*) and the endogenous control HPRT1 (hypoxanthine phosphoribosyltransferase 1) were purchased as TaqMan^® ^Gene Expression Assays (Applied Biosystems). The assay numbers for these genes were as follows: Hs00201046_m1 (HYAL1); Hs00186841_m1 (HYAL2); Hs00758053_m1 (HAS1); Hs00193435_m1 (HAS2); Hs00193436_m1 (HAS3); Hs99999909_m1 (HPRT). The assays were supplied as a 20× Mix of PCR primers and TaqMan MGB (minor groove binder) probes labeled with a 6-FAM dye and a non-fluorescent quencher at the 3' end of the probe. The primers were designed to span an exon-exon junction, eliminating the possibility of detecting genomic DNA.

For each amplification, 6 μl of cDNA equivalent to 30 ng of total RNA was mixed with 1 μl of 20 × Primer and Probe Mix and 10 μl of 2 × TaqMan Universal Master Mix in a final volume of 20 μl. Each sample was quantified using standard curves that were established by 6 series of 4-fold serial dilution of cDNA obtained by reverse transcription of 2.5 μg Universal Human Reference RNA (Stratagene, La Jolla, CA). The standard curves and no-template negative controls (NTC) were made on every plate. Each sample and each point of the standard curve was performed in triplicate reactions. The reactions were performed in 96-well plates on the MX3000P real-time instrument (Stratagene, La Jolla, CA). The PCR conditions were as follows: 1 cycle at 95°C for 10 min, followed by 40 cycles of 95°C for 15 sec, 60°C for 1 min.

The *HPRT1 *gene we used for normalization is an accurate reference for the quantitative gene expression assays in clinical tumor samples [19]. Relative gene expression values were calculated as the ratio between the target gene and *HPRT1*, obtained for each sample from the standard curves. Finally, these values were divided by the mean value of normal ovaries. C_T _values were used to roughly compare the relative amounts of *HYAL1 *and *HYAL2 *mRNAs.

### Hyaluronidase assay

Hyaluronidase activities in the tissue extracts were determined by the release of biotinylated hyaluronan coupled to the bottom of 96-well plates in triplicate reactions, as previously described. The results were normalized to protein concentrations in the tissue extracts [3].

### Staining of Hyaluronan

Deparaffinized 5-μm tissue sections were stained for hyaluronan with our own preparation of biotinylated hyaluronan-binding complex (bHABC) as described in detail previously [1]. All samples were scored by an observer unaware of the clinical data (M.A.) The percentage of tumor area with the strong intensity was estimated using a continuous scale (0–100%), and used as an indicator of hyaluronan accumulation.

### Staining of HASs

Antigen retrieval was performed for HAS2 staining by microwave treatment (700 W, 3 × 5 min) in citrate buffer. Thereafter all deparaffinized sections were treated for 5 min with 1% H_2_O_2 _to block endogenous peroxidase, washed with PB, and incubated in 1% bovine serum albumin (BSA) in PB for 30 min to block nonspecific binding. Thereafter the sections were incubated overnight at 4°C with polyclonal antibodies for HAS1 (1:100 dilution in 1% BSA, Santa Cruz Biotechnology, inc., Santa Cruz, CA), HAS2 (1:50, Santa Cruz) or HAS3 (1:100, Santa Cruz), followed by a 1 h incubation with biotinylated antigoat antibody (1:1000, Vector Laboratories). The bound antibodies were visualized with the avidin-biotin peroxidase method (1:200, Vectastain Kit, Vector Laboratories). The sections were incubated for 5 min in 0.05% diaminobenzidine (Sigma) and 0.03% hydrogen peroxide in PB. After washes, the sections were counterstained with Mayer's hematoxylin for 1 min, washed, dehydrated, and mounted in DPX (Gurr, BDH Laboratory Supplies, Poole, U.K.). The percentage of positive area for each HAS was estimated in stroma and in epithelium for HAS1 and HAS3. Staining intensity positivity of HAS2 in the epithelium was estimated grading it into three categories: 1 (weak); 2 (moderate); and 3 (strong).

### Statistical methods

Statistical analyses were carried out using SPSS 11.5 for Windows (SPSS, Chigago, IL). Differences between groups were first analysed by non-parametric Kruskal-Wallis test, and when found significant were followed by non-parametric Mann – Whitney U-test for paired comparisons between the patient groups. Also Chi-square test were used to evaluate HAS2 epithelial staining. Correlations between *HAS3*, *HYAL1 *and *HYAL2 *gene expression, hyaluronidase activity, hyaluronan and HAS stainings were analysed by the Spearman's correlation test.

## Results

### Hyaluronan content

**Figure 1:**
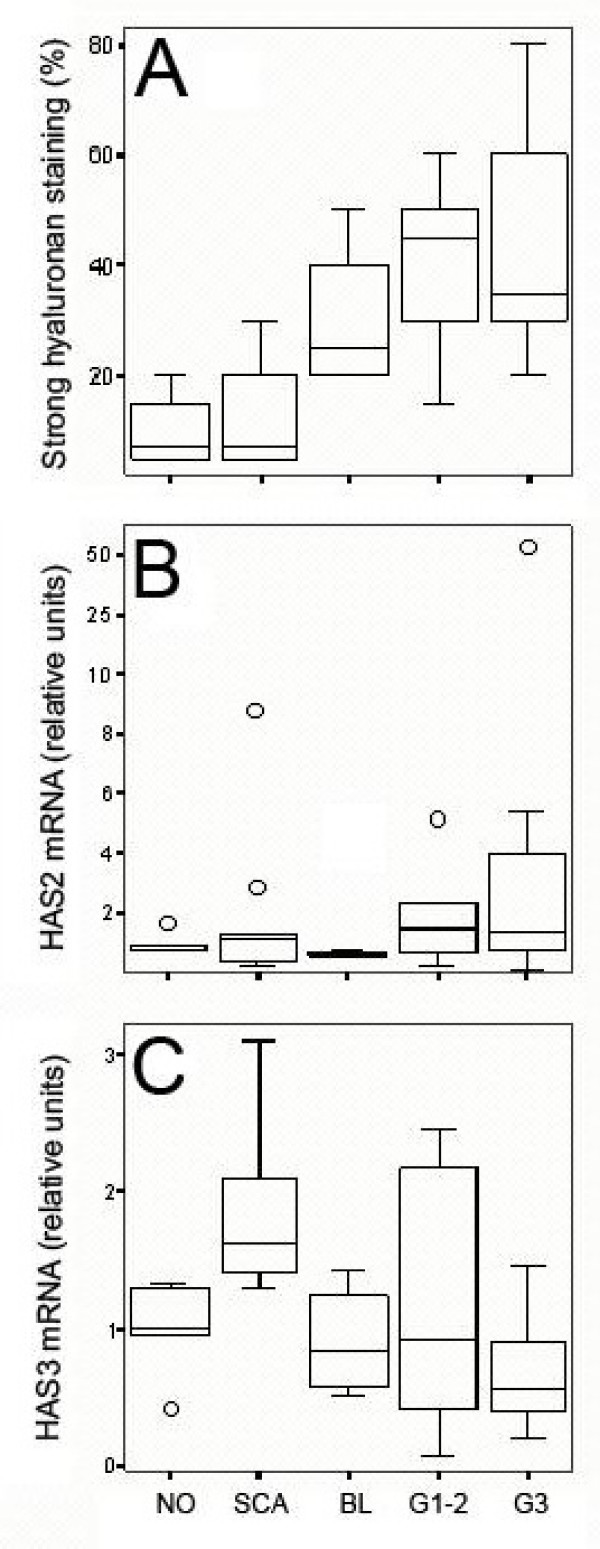
**Hyaluronan content and HAS expression in human ovary and its tumors**. A) Strong hyaluronan staining intensity (%) in NO (n = 4), SCA (n = 10), BL (n = 4), G1–2 (n = 10) and G3 (n = 10) tissues. Relative mRNA levels of B) HAS2 and C) HAS3 in NO (n = 5), SCA (n = 10), BL (n = 4), G1+2 (n = 10) and G3 (n = 10) tissues. The boxes show the ranges between 25th and 75th percentiles, with a horizontal line at the median value. The whiskers extend to the 10th and 90th percentiles. The open circles represent the statistical outlier values. NO = normal ovary, SCA = serous cystadenoma, BL = serous borderline tumor, G1–2 = grade 1–2 serous cystadenocarcinoma, G3 = grade 3 serous cystadenocarcinoma.

The level of hyaluronan accumulation in the present set of tumors was scored from tissue sections using a biotinylated probe that specifically binds hyaluronan [1]. This histological assay closely correlates with biochemical quantitation of hyaluronan [3]. The content of hyaluronan in the benign tumors was close to that of the normal ovary, while the malignant tumors showed markedly increased levels (malignant tumors vs. other lesions, P = 0.00026) (Fig. 1A).

### Expression of *HAS1 *and *HAS2*

Since increased synthesis of hyaluronan often accounts for hyaluronan accumulation, and since transcriptional regulation seems to dominate the synthesis, mRNA from these samples was analyzed by real-time RT-PCR for the hyaluronan synthases *HAS1*, *HAS2*, and *HAS3*. Transcripts of *HAS1 *were detected at such a low level that reliable quantitation was not possible. The expression of *HAS2 *was not markedly changed in benign and borderline tumors (Fig. 1B). The median of *HAS2 *mRNA was 51–61% higher in the malignant tumors, compared with normal ovaries, but the variance between individual tumors was extensive (Fig. 1B). Overall, there were no statistically significant differences in HAS2 between the groups (P = 0.387).

### Expression of *HAS3*

The expression of *HAS3 *was more consistent, with significant differences between the subgroups (Kruskal-Wallis test; P = 0.0084) (Fig 1C). Groupwise testing showed increased *HAS3 *expression in benign tumors compared to normal ovaries (median +60%, P = 0.0039). As compared with normal ovaries, the median values of *HAS3 *in borderline and grade 1+2 malignant tumors were not changed, while grade 3 showed a trend for decline (-44%) (Fig. 1C).

### Hyaluronidase activity

**Figure 2:**
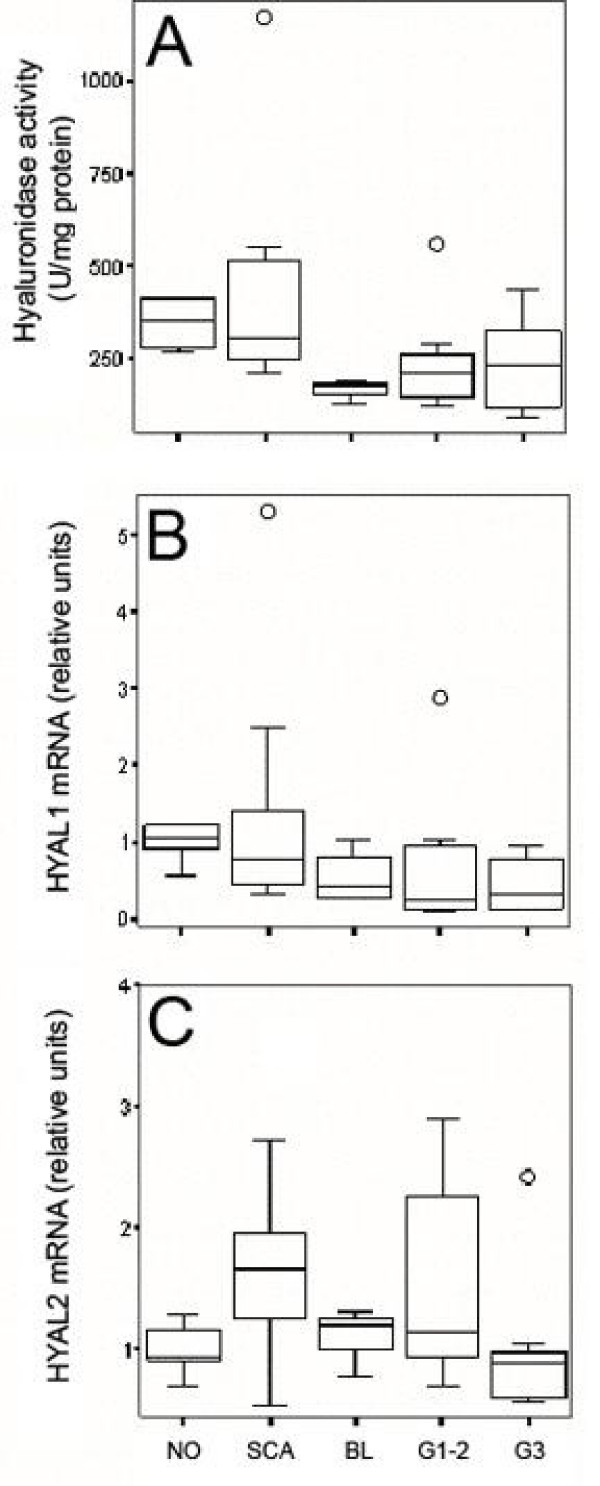
**Hyaluronidase activity and HYAL1–2 expression in human ovary and its tumors**. A) Hyaluronidase activity in NO (n = 4), SCA (n = 8), BL (n = 3), G1–2 (n = 8) and G3 (n = 9) tissues. Relative mRNA levels of B) HYAL1 and C) HYAL2 in NO (n = 5), SCA (n = 10), BL (n = 4), G1–2 (n = 10) and G3 (n = 10). The boxes show the ranges between 25th and 75th percentiles, with a horizontal line at the median value. The whiskers extend to the 10th and 90th percentiles. The open circles represent the statistical outlier values. NO = normal ovary, SCA = serous cystadenoma, BL = serous borderline tumor, G1–2 = grade 1–2 serous cystadenocarcinoma, G3 = grade 3 serous cystadenocarcinoma.

The somewhat unexpected finding that the levels of *HAS *mRNA were not consistently elevated in the tumors, turned our attention to hyaluronidases. As noted before, there is hyaluronidase activity at pH 3.7 in ovarian tissues, with a tendency to decrease in malignant tumors [3]. In the present material, the median values of hyaluronidase activity in borderline and malignant tumors were indeed 58-40% lower than in normal ovary, but the difference did not reach statistical significance (P = 0.076) (Fig. 2A).

### Expression of *HYAL1*

Since two ubiquitous hyaluronidases, *HYAL1 *and *HYAL2 *were likely to account for the hyaluronidase activity, we quantified their mRNA levels by real-time RT-PCR. There was a gradual decline in *HYAL1 *expression from normal ovaries through benign and borderline tumors down to the cancers (Fig. 2B), with statistically significant differences between the groups (P = 0.022). A groupwise analysis indicated decreased *HYAL1 *in all non-benign tumors compared to normal ovaries (borderline: -58% (median), P = 0.05; grades 1+2: -79%, P = 0.05; grade 3: -69%, P = 0.01). The malignant grade 1+2 and grade 3 tumors expressed also significantly less *HYAL1 *than benign tumors (P = 0.034 and P = 0.028, respectively) (Fig. 2B).

### Expression of HYAL2

The Kruskall-Wallis test indicated significant differences also between the groups in *HYAL2 *expression (P = 0.033). An increase of *HYAL2 *expression occurred in benign tumors compared to normal ovaries (+76%, P = 0.037), and while a decrease was noted in grade 3 cancers compared to benign tumors (P = 0.0156) (Fig. 2C).

### Relationship between hyaluronan accumulation and *HAS *and *HYAL *mRNA levels

No significant correlations were found between the level of HAS2 or HAS3 mRNA and the hyaluronan content. Instead, hyaluronidase activity showed a significant inverse correlation with hyaluronan content (r = -0.5; P = 0.003). Furthermore, *HYAL1 *transcript levels correlated with hyaluronan content (r = -0.4; P = 0.025) and hyaluronidase activity (r = 0.5; P = 0.006, n = 32), suggesting that *HYAL1 *dominated the differences in hyaluronidase activity and contributed to the accumulation of hyaluronan in the ovarian cancers.

Interestingly, *HYAL2 *did not correlate with hyaluronidase activity, even though its mRNA level was 2–3 orders of magnitude higher than that of *HYAL1*, as suggested by the real-time RT-PCR assay. Despite their apparently divergent changes in different tumor types, *HYAL2 *and *HYAL1 *mRNA levels still correlated positively with each other in the whole patient material (r = 0.5; P = 0.0013).

### HAS immunostainings

**Figure 3:**
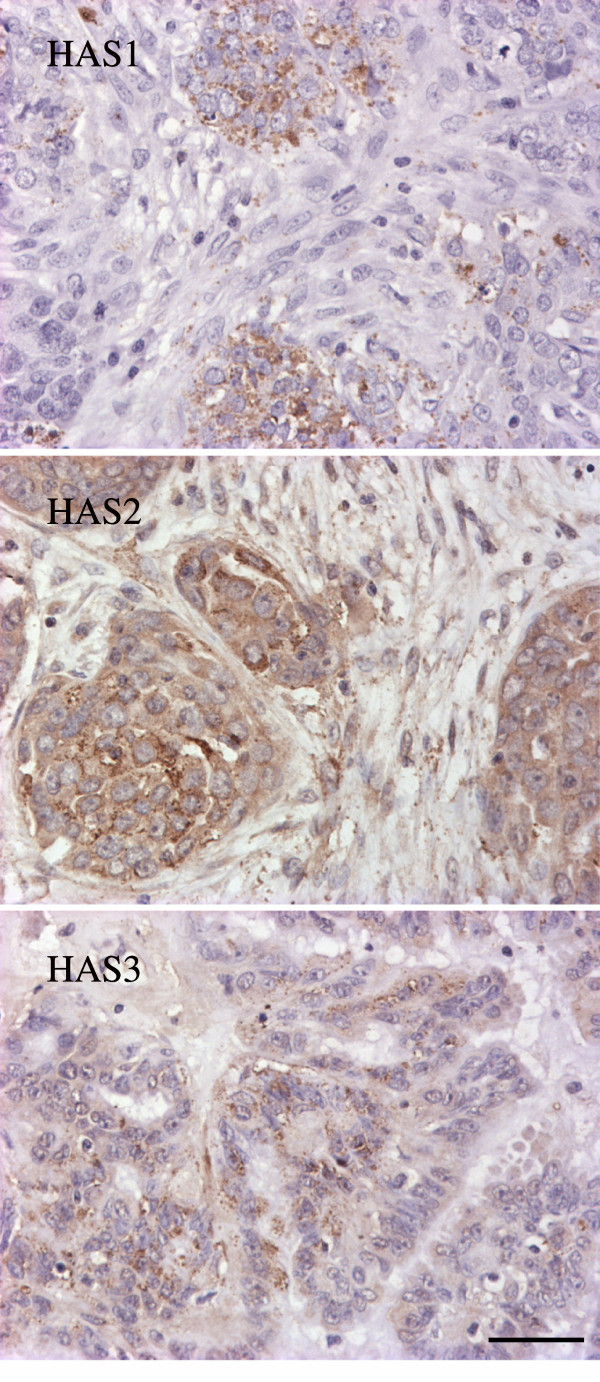
**Distribution of HAS immunoreactivity in ovarian cancer**. Sections of ovarian cancer tissues were stained with antibodies against HAS1, HAS2 and HAS3, as indicated in the panels. The brown color indicates HAS, nuclei are stained with haematoxylin (blue). Note that HAS1 and HAS3 immunoreactivities reside mostly in the nests of the tumor epithelial cells, while HAS2 is present also in the stromal cells. Scale bar 50 μm.

HAS1-positive cells were not present in any of the normal specimens or in the stroma of cancers, but 41% of the cancer samples had a low percentage of HAS1 positive cancer epithelial cells (Fig. 3) (Table 2). The percentage of the HAS1 positive cells did not correlate with the mRNA levels, hyaluronan or with the histological groups or grades.

**Table 2:** Immunostaining of HAS1, HAS3 and HAS2 in human ovary and its tumors

	HAS1 staining intensity*		HAS3 staining intensity*		HAS2 staining intensity			
					
	Stroma	Epithelium	Stroma	Epithelium	Stroma †	Epithelium ‡		
						weak	moderate	strong
						
Normal Ovary (n = 5)	0	0	0	0	37 (10 – 50%)	5	0	0
Benign (n = 10)	0	4 (1–5%)	0	4 (1–5%)	31 (10 – 50%)	0	5	5
Borderline (n = 4)	0	2 (5%)	0	1 (10%)	24 (10 – 25%)	1	2	1
Grade 1 + 2 (n = 10)	0	7 (5–50%)	0	6 (5–50%)	13 (10 – 25%)	3	4	3
Grade 3 (n = 10)	0	4 (1–10%)	0	7 (1–10%)	25 (10 – 50%)	6	4	0

Benign = serous cystadenoma, Borderline = serous borderline tumor

Grade 1–3 = serous epithelial cystadenocarcinoma

* num. of positive samples (% of positive cells), † mean % of positive cells (range), ‡ distripution of staining intensity

Normal specimens showed no HAS3 signal but 46% of the cancers presented generally low numbers of HAS3-positive cancer epithelial cells (Fig. 3) (Table 2). An analysis including both normal and different tumor specimens indicated that the proportion (%) of the HAS3-positive cells of all epithelial cells correlated with hyaluronan staining in the stroma (r = 0.424, p = 0.008), and negatively with HYAL1 mRNA (r = -0.438, p = 0.005). HAS3 immunostainings did not correlate with the histological type of specimens, or with tumor grade.

The anti-HAS2-antibody showed a more widespread staining and, in contrast to HAS1 and HAS3 antibodies, it stained both epithelial and stromal cells (Fig. 3) (Table 2). In normal ovaries all epithelial cells showed weak HAS2 staining (Table 2). All tumor samples also showed HAS2 signal in the cancer epithelial cells, but the staining intensity was more variable. In addition to samples with weakly stained epithelial tissue similar to normal ovaries, 64% of tumor samples showed a more intense epithelial HAS2-immunostaining. The highest HAS2 staining intensities were detected in benign tumors and in grade 1 carcinomas (Table 2) and the intensities (graded into three categories) was significantly different in the histological subgroups (Chi-square P = 0.003). Of the stromal cells, 25–37% were HAS2 positive both in normal ovaries and in tumor specimens (Fig. 3) (Table 1) but the proportion of HAS2-positive stromal cells did not correlate either with *HAS2 *mRNA level, hyaluronan staining intensity or histological groups.

## Discussion

Hyaluronan synthase (*HAS*) and hyaluronidase mRNA levels were quantitated for the first time in ovarian cancers and normal ovaries and benign tumors, and the results correlated with hyaluronan and HAS immunocytochemistry in the corresponding tissue sections.

There was little *HAS1 *mRNA, no consistent upregulation of *HAS2 *in the cancers, and the median values of *HAS3 *mRNA were actually lower in cancers than controls. Immunohistochemical stainings of HAS proteins showed a low level of HAS1, a slightly elevated level of HAS3 in the tumor epithelia, and a variable elevation of HAS2 immunostaining in the tumor epithelial cells, in agreement with the mRNA assays. In the stromal cells, no difference was observed with the HAS2 antibody in normal and tumor samples, an unexpected result considering the strong accumulation of hyaluronan in the same specimens. Instead, *HYAL1 *expression was consistently decreased in the cancers, with a concomitant trend to reduced hyaluronidase enzyme activity.

### HAS expression in ovarian cancer

Since *HAS2 *and *HAS3 *genes showed no consistent increase in their expression in the serous ovarian cancers, and *HAS1 *mRNA was virtually absent, changes in the transcriptional activity of the *HAS *genes seem not to be the main factor in the increased hyaluronan content of these tumors. A few of the samples showed high expression levels of *HAS2*, but most of the cancers showed no elevation in the expression of any of the *HAS *genes.

Immunohistochemical stainings confirmed that the levels of HAS1 and HAS3 were relatively low in ovarian cancers, while the signal for HAS2 was more widespread, in line with the real-time RT-PCR-analysis. While the stromal cells were positive for HAS2, their staining intensity did not correlate with that of stromal hyaluronan, nor with the tumor type or grade. Unexpectedly, the HAS2 antibody stained also normal, benign and malignant ovarian epithelial cells, all of which were largely negative with the hyaluronan probe. Putting these findings together would suggest that the epithelium somehow contributes to hyaluronan mainly seen in the stroma. Theoretically, this would be possible if the epithelial cells were unable to hold and take up the synthesized hyaluronan by a receptor like CD44. In support of this idea, the expression of CD44 is reduced in the high grade ovarian cancers [20], and the released hyaluronan could be trapped in the stroma by complexing with versican [21]. Even if the epithelial HAS contributes to stromal hyaluronan, it would not explain the hyaluronan accumulation in the high grade tumors since the epithelial HAS2 staining intensity was highest in the benign and well differentiated tumors.

Taken together, the data suggest that although in some of the ovarian tumors a high HAS2/HAS3 level may contribute to hyaluronan accumulation, in the majority of cases, particularly the high grade tumors, stromal hyaluronan accumulation is not explained by the increased expression of any of the HASs.

### Relationship between HYAL1 and HYAL2

In terms of the cellular content of mRNA, the dominant hyaluronidase in these tissues was *HYAL2*. However, the fact that only *HYAL1 *mRNA correlated with the measured hyaluronidase activity, and inversely with hyaluronan accumulation, suggests higher enzymatic activity of HYAL1 and more importance in hyaluronan catabolism. It has been noted that HYAL2 preferentially produces hyaluronan fragments above 20 kDa, while *HYAL1 *can cut hyaluronan down to tetra- hexasaccharides [22], suggesting that they either act in sequence in the degradation, or have distinct functions related to the sizes of the end products [9].

There was also a trend for low *HYAL2 *expression in the most aggressive, grade 3 tumors, a result resembling that in diffuse large B-cell lymphomas (DLBCLs) [23]. Like in ovarian cancer, hyaluronidase activity in DCBCL tissue extracts was not correlated with *HYAL2 *expression [23]. However, there was an inverse correlation between *HYAL2 *expression and hyaluronan content [23]. Obviously, we need to know more about the distinct cellular functions of *HYAL1 *and *HYAL2 *to define their exact roles in malignancies.

### Genomic changes in the HYAL genes

Allelic imbalance is frequent in the 3p21.3 chromosome region containing *HYAL1 *and *HYAL2 *genes, suggesting that this site is important in ovarian [24] and other cancers [9,11]. The positive correlation that still existed between the expression of *HYAL1 *and *HYAL2 *may be explained by concomitant deletions of these closely mapped genes. Whether due to genomic alterations or not, the present results suggest that hyaluronidase activity contributes to the accumulation of hyaluronan, a known promoter of malignant growth.

### HYAL1 changes in ovarian vs. other cancers

The present findings of reduced *HYAL1 *expression are consistent with those of another set of ovarian cancer samples [3], but in a strong contrast to reports on prostate and bladder tumors in which increased *HYAL1 *expression in poorly differentiated tumors is associated with advanced disease and unfavorable prognosis [15,16]. It appears that malignancies arising from different cell types utilize distinct strategies to survive and progress. Increased hyaluronan may contribute to tumor growth and invasiveness by providing an expanded, loose matrix for cancer cells, protecting the tumor from immune reactions and apoptosis, stimulating tumor cell migration, and increasing cell proliferation [4]. While hyaluronidase may block the above functions associated with high molecular mass hyaluronan, it can at the same time create hyaluronan oligosaccharides that stimulate neovascularization [25], a crucial precondition for tumor expansion. The relative importance of the opposite roles of hyaluronidase function in a particular type of cancer probably determines the outcome. The exact expression level is also important; transfections of *HYAL1 *can either promote or suppress malignant growth in a single cell type, depending on the resulting enzyme activity [16].

### Possible mechanisms of HYAL-1 tumor suppressor function

High HYAL1 expression can result in apoptosis by increasing the expression of WOX1 (WW domain-containing oxidoreductase, WWOX) [16]. WOX1 causes mitochondrial permeabilization and is an essential partner of p53 in cell death [26]. Importantly, WWOX variant 1 expression is significantly lower in ovarian epithelial tumours than in normal ovaries, which supports its role as a suppressor of ovarian tumorigenesis [27]. Hyaluronidase can also cause apoptosis by inducing NAD+-linked 15-hydroxyprostaglandin dehydrogenase (15-PDGH), an enzyme that degrades prostaglandins and promotes apoptosis in lung carcinoma cells [28]. Furthermore, the high molecular mass hyaluronan that occupies cell surface CD44 receptors maintains p-Akt and PI3K dependent signals that prevent cancer cell apoptosis, while hyaluronidase, and the oligosaccharides created by hyaluronidase, block these cell survival signals [29].

## Conclusion

In conclusion, our results indicate for the first time that decreased expression of HYAL1 rather than increased expression of HAS1–3 correlates with the accumulation of hyaluronan in serous ovarian cancer, and provides new insight in the role of hyaluronidases in human cancer *in vivo*.

## Abbreviations

HYAL: hyaluronidase; HAS: hyaluronan synthase; FIGO: International Federation of Gynecologists and Obstetrics.

## Competing interests

The authors declare that they have no competing interests.

## Authors' contributions

TN performed the RNA extraction and RT-QPCR analysis, carried out hyaluronidase assays, performed statistical analysis and drafted the manuscript. KR analyzed the HAS staining and contributed to the manuscript. RS contributed to the manuscript and participated to the design of the RT-QPCR analysis. MIT, RT and VMK participated in design of the study and contributed to the manuscript. KH contributed to pathological analysis of the tissue samples. AMH and MK participated in tumor sample collection. MA conceived of the study, and participated in its design and coordination and helped to draft the manuscript

## Pre-publication history

The pre-publication history for this paper can be accessed here:

http://www.biomedcentral.com/1471-2407/9/143/prepub

## References

[B1] Anttila MA Tammi RH Tammi MI Syrjanen KJ Saarikoski SV Kosma VM High levels of stromal hyaluronan predict poor disease outcome in epithelial ovarian cancer Cancer Res 2000 60 150 5 10646867

[B2] Strobel T Swanson L Cannistra SA In vivo inhibition of CD44 limits intra-abdominal spread of a human ovarian cancer xenograft in nude mice: A novel role for CD44 in the process of peritoneal implantation Cancer Res 1997 57 1228 32 9102203

[B3] Hiltunen EL Anttila M Kultti A Ropponen K Penttinen J Yliskoski M Kuronen AT Juhola M Tammi R Tammi M Kosma VM Elevated hyaluronan concentration without hyaluronidase activation in malignant epithelial ovarian tumors Cancer Res 2002 62 6410 3 12438225

[B4] Toole BP Wight TN Tammi MI Hyaluronan-cell interactions in cancer and vascular disease J Biol Chem 2002 277 4593 6 10.1074/jbc.R100039200 11717318

[B5] Weigel PH Hascall VC Tammi M Hyaluronan synthases J Biol Chem 1997 272 13997 4000 10.1074/jbc.272.22.13997 9206724

[B6] Jacobson A Rahmanian M Rubin K Heldin P Expression of hyaluronan synthase 2 or hyaluronidase 1 differentially affect the growth rate of transplantable colon carcinoma cell tumors Int J Cancer 2002 102 212 9 10.1002/ijc.10683 12397638

[B7] Golshani R Lopez L Estrella V Kramer M Iida N Lokeshwar VB Hyaluronic acid synthase-1 expression regulates bladder cancer growth, invasion, and angiogenesis through CD44 Cancer Res 2008 68 483 91 10.1158/0008-5472.CAN-07-2140 18199543

[B8] Bharadwaj AG Kovar JL Loughman E Elowsky C Oakley GG Simpson MA Spontaneous metastasis of prostate cancer is promoted by excess hyaluronan synthesis and processing Am J Pathol 2009 174 1027 36 10.2353/ajpath.2009.080501 19218337 PMC2665762

[B9] Stern R Hyaluronan metabolism: A major paradox in cancer biology Pathol Biol (Paris) 2005 53 372 82 16085113 10.1016/j.patbio.2004.12.021

[B10] Tammi R Rilla K Pienimaki JP MacCallum DK Hogg M Luukkonen M Hascall VC Tammi M Hyaluronan enters keratinocytes by a novel endocytic route for catabolism J Biol Chem 2001 276 35111 22 10.1074/jbc.M103481200 11451952

[B11] Csoka AB Frost GI Stern R The six hyaluronidase-like genes in the human and mouse genomes Matrix Biol 2001 20 499 508 10.1016/S0945-053X(01)00172-X 11731267

[B12] Hemming R Martin DC Slominski E Nagy JI Halayko AJ Pind S Triggs-Raine B Mouse Hyal3 encodes a 45- to 56-kDa glycoprotein whose overexpression increases hyaluronidase 1 activity in cultured cells Glycobiology 2008 18 280 9 10.1093/glycob/cwn006 18234732

[B13] Atmuri V Martin DC Hemming R Gutsol A Byers S Sahebjam S Thliveris JA Mort JS Carmona E Anderson JE Dakshinamurti S Triggs-Raine B Hyaluronidase 3 (HYAL3) knockout mice do not display evidence of hyaluronan accumulation Matrix Biol 2008 27 653 60 10.1016/j.matbio.2008.07.006 18762256

[B14] Liu D Pearlman E Diaconu E Guo K Mori H Haqqi T Markowitz S Willson J Sy MS Expression of hyaluronidase by tumor cells induces angiogenesis in vivo Proc Natl Acad Sci USA 1996 93 7832 7 8755562 10.1073/pnas.93.15.7832 PMC38834

[B15] Lokeshwar VB Cerwinka WH Lokeshwar BL HYAL1 hyaluronidase: A molecular determinant of bladder tumor growth and invasion Cancer Res 2005 65 2243 50 10.1158/0008-5472.CAN-04-2805 15781637

[B16] Lokeshwar VB Cerwinka WH Isoyama T Lokeshwar BL HYAL1 hyaluronidase in prostate cancer: A tumor promoter and suppressor Cancer Res 2005 65 7782 9 10.1158/0008-5472.CAN-04-2805 16140946

[B17] Bertrand P Girard N Duval C d'Anjou J Chauzy C Menard JF Delpech B Increased hyaluronidase levels in breast tumor metastases Int J Cancer 1997 73 327 31 10.1002/(SICI)1097-0215(19971104)73:3<327::AID-IJC4>3.0.CO;2-1 9359477

[B18] Karseladze AI WHO histological classification of ovarian tumors Geneva, 1999 (R.E.scully, L.H.sobin. Arkh Patol 2005 Suppl 1 64 16108150

[B19] de Kok JB Roelofs RW Giesendorf BA Pennings JL Waas ET Feuth T Swinkels DW Span PN Normalization of gene expression measurements in tumor tissues: Comparison of 13 endogenous control genes Lab Invest 2005 85 154 9 15543203 10.1038/labinvest.3700208

[B20] Sillanpää S Anttila MA Voutilainen K Tammi RH Tammi MI Saarikoski SV Kosma VM CD44 expression indicates favorable prognosis in epithelial ovarian cancer Clin Cancer Res 2003 9 5318 24 14614016

[B21] Voutilainen K Anttila M Sillanpää S Tammi R Tammi M Saarikoski S Kosma VM Versican in epithelial ovarian cancer: relation to hyaluronan, clinicopathologic factors and prognosis Int J Cancer 2003 107 359 64 10.1002/ijc.11423 14506734

[B22] Lepperdinger G Mullegger J Kreil G Hyal2 – less active, but more versatile? Matrix Biol 2001 20 509 14 10.1016/S0945-053X(01)00170-6 11731268

[B23] Bertrand P Courel MN Maingonnat C Jardin F Tilly H Bastard C Expression of HYAL2 mRNA, hyaluronan and hyaluronidase in B-cell non-hodgkin lymphoma: Relationship with tumor aggressiveness Int J Cancer 2005 113 207 12 10.1002/ijc.20562 15386412

[B24] Tuhkanen H Anttila M Kosma VM Genetic alterations in the peritumoral stromal cells of malignant and borderline epithelial ovarian tumors as indicated by allelic imbalance on chromosome 3p Int J Cancer 2004 109 247 52 10.1002/ijc.11733 14750176

[B25] Slevin M Krupinski J Kumar S Gaffney J Angiogenic oligosaccharides of hyaluronan induce protein tyrosine kinase activity in endothelial cells and activate a cytoplasmic signal transduction pathway resulting in proliferation Lab Invest 1998 78 987 1003 9714186

[B26] Chang NS Pratt N Heath J Hyaluronidase induction of a WW domain-containing oxidoreductase that enhances tumor necrosis factor cytotoxicity J Biol Chem 2001 276 3361 70 10.1074/jbc.M007140200 11058590

[B27] Gourley C Paige AJ Taylor KJ WWOX mRNA expression profile in epithelial ovarian cancer supports the role of WWOX variant 1 as a tumour suppressor, although the role of variant 4 remains unclear Int J Oncol 2005 26 1681 9 15870886 10.3892/ijo.26.6.1681 PMC4166600

[B28] Ding Y Tong M Liu S Moscow JA Tai HH NAD+-linked 15-hydroxyprostaglandin dehydrogenase (15-PGDH) behaves as a tumor suppressor in lung cancer Carcinogenesis 2005 26 65 72 10.1093/carcin/bgh277 15358636

[B29] Ghatak S Misra S Toole BP Hyaluronan oligosaccharides inhibit anchorage-independent growth of tumor cells by suppressing the phosphoinositide 3-kinase/Akt cell survival pathway J Biol Chem 2002 11 38013 20 10.1074/jbc.M202404200 12145277

